# Overexpression of the Transcription Factor *AtLEC1* Significantly Improved the Lipid Content of *Chlorella ellipsoidea*

**DOI:** 10.3389/fbioe.2021.626162

**Published:** 2021-02-17

**Authors:** Xiao Liu, Dan Zhang, Jianhui Zhang, Yuhong Chen, Xiuli Liu, Chengming Fan, Richard R-C. Wang, Yongyue Hou, Zanmin Hu

**Affiliations:** ^1^State Key Laboratory of Plant Cell and Chromosome Engineering, Institute of Genetics and Developmental Biology, Innovation Academy for Seed Design, Chinese Academy of Sciences, Beijing, China; ^2^Analysis and Test Center, Guangzhou Higher Education Mega Center, Guangdong University of Technology, Guangzhou, China; ^3^Inner Mongolia Academy of Agriculture and Animal Husbandry, Huhhot, China; ^4^United States Department of Agriculture, Agricultural Research Service, Forage and Range Research Laboratory, Utah State University, Logan, UT, United States; ^5^College of Agriculture, University of Chinese Academy of Sciences, Beijing, China

**Keywords:** *Chlorella ellipsoidea*, *AtLEC1*, lipid content, transcriptome, regulation

## Abstract

Microalgae are considered to be a highly promising source for the production of biodiesel. However, the regulatory mechanism governing lipid biosynthesis has not been fully elucidated to date, and the improvement of lipid accumulation in microalgae is essential for the effective production of biodiesel. In this study, *LEAFY COTYLEDON1 (LEC1)* from *Arabidopsis thaliana*, a transcription factor (TF) that affects lipid content, was transferred into *Chlorella ellipsoidea*. Compared with wild-type (WT) strains, the total fatty acid content and total lipid content of *AtLEC1* transgenic strains were significantly increased by 24.20–32.65 and 22.14–29.91%, respectively, under mixotrophic culture conditions and increased by 24.4–28.87 and 21.69–30.45%, respectively, under autotrophic conditions, while the protein content of the transgenic strains was significantly decreased by 18.23–21.44 and 12.28–18.66%, respectively, under mixotrophic and autotrophic conditions. Fortunately, the lipid and protein content variation did not affect the growth rate and biomass of transgenic strains under the two culture conditions. According to the transcriptomic data, the expression of 924 genes was significantly changed in the transgenic strain (LEC1-1). Of the 924 genes, 360 were upregulated, and 564 were downregulated. Based on qRT-PCR results, the expression profiles of key genes in the lipid synthesis pathway, such as *ACCase*, *GPDH*, *PDAT1*, and *DGAT1*, were significantly changed. By comparing the differentially expressed genes (DEGs) regulated by *AtLEC1* in *C. ellipsoidea* and *Arabidopsis*, we observed that approximately 59% (95/160) of the genes related to lipid metabolism were upregulated in *AtLEC1* transgenic *Chlorella*. Our research provides a means of increasing lipid content by introducing exogenous TF and presents a possible mechanism of *AtLEC1* regulation of lipid accumulation in *C. ellipsoidea*.

## Introduction

The sustainable development of biofuels has gained considerable attention in recent years (Koutra et al., 2018). Microalgae biomass and the energy-rich compounds derived from microalgae, such as carbohydrates and lipids, have emerged as the most popular feedstock for the production of biofuels (Wijffels and Barbosa, 2010; Du et al., 2019). The first- and second-generation biofuel feedstock, such as palm, soya beans, rapeseed and wheat, had the disadvantage that the cultivation of these crops might compete for limited arable farmland, which indirectly affects food security and prices (Zhu et al., 2016; Park et al., 2019).

Furthermore, compared with other biofuel feedstocks and terrestrial plants, microalgae are more appropriate for biofuel production because (1) as photosynthetic organisms, microalgae are able to capture solar energy and use water and atmospheric CO_2_ with high efficiency to accumulate biomass in the form of organic ingredients, such as lipids (Hu et al., 2008); (2) they could grow in seawater or industrial/domestic wastewaters with a relatively high growth rate (Venkata et al., 2015); and (3) microalgae are environmentally friendly resources for biomass energy, and they could reduce the greenhouse gas effect (Chisti, 2007). However, several factors may limit stable production of microalgae: (1) it is difficult to select appropriate strains that could produce on a large scale and contain high levels of lipids (Xiong et al., 2010); and (2) environmental factors, such as light, temperature, pH, available nutrients, and higher cost of cultivation, restrict microalgae production (Shin et al., 2018).

Some microalgae species, such as *Botryococcus braunii* (57–64%), *Schizochytrium* sp. (50–77%), and *Neochloris oleoabundans* (35–65%), have a high lipid content but slow growth rate and low oil production rates (Rao et al., 2007). However, several other species, such as *Chlamydomonas reinhardtii*, *Chlorella pyrenoidosa*, and *Navicula pelliculosa*, have a high growth rate but a low oil content (<15%) (Hu et al., 2008). Thus, it appears to be difficult to locate the microalgae with simultaneous high cell growth rate and high cellular lipid content. Many efforts have been made to overcome these challenges, such as strain selection (Remmers et al., 2018), the improvement of culture nutrition (especially N, P, and S limitation), and other improvements in growth conditions (temperature, light, and pH) (Guschina and Harwood, 2006; Markou and Nerantzis, 2013; Li-Beisson et al., 2019).

Recently, the rapid development of multiple approaches, including omic analysis, genetic engineering, genome editing, and metabolic pathway engineering, provided efficient ways to increase the lipid content in microalgae. Omics analyses identified complete gene sets encoding fatty acid and triacylglyceride biosynthetic pathways of *Chlorella vulgaris* UTEX 395 (Guarnieri et al., 2018). Compartmentalized genome scale metabolic model iAJ526 was reconstructed with 1,455 reactions, 1,236 metabolites, and 526 genes for *Chlorella variabilis* (Juneja et al., 2016). Proteomic analysis of *C. vulgaris* showed the mechanisms governing lipid accumulation in algae (Guarnieri et al., 2013). Fan et al. sequenced the 56.8-Mbp genome of *C. pyrenoidosa* FACHB-9 to investigate the rapid switch of the intracellular energy storage form from starch to lipids and showed that overexpression of an NAD(H) kinase from *Arabidopsis* increased cellular lipid content by 110.4% (Fan et al., 2015). Chakraborty et al. (2016) found nitrate limitation (1 mM) was suitable for the enhancement of lipids, resulting in the highest yield (48.26% w/w) by using the Taguchi model. Ma et al. (2019) reported that overexpressing ACCase and PEPC genes in a lipid-poor wild strain MC-1 could increase lipid content by 28.6%. In 2014, the CRISPR/Cas9 system was reported to have worked successfully in *C. reinhardtii* (Jiang et al., 2014). Lin and Ng used CRISPR/Cas9 to edit the *fad3* gene and achieved an accumulation of lipid content higher by 46% (w/w) in *C. vulgaris* FSP-E (Lin and Ng, 2020).

Clearly, the first step of the lipid biosynthesis, acetyl-CoA carboxylase (ACCase), plays a vital role in metabolic flux to lipid biosynthesis, since ACC catalyzes the carboxylation of acetyl-CoA to form malonyl-CoA, the first intermediate product in the fatty acid elongation pathway (Kim, 1997; Davis et al., 2000). Next, a series of reactions for fatty acid production are catalyzed by fatty acid synthase (FAS) (Subrahmanyam and Cronan, 1998). However, lipid synthesis and accumulation are controlled by multiple genes. A number of studies show transcription factors (TFs) that regulate multiple genes play an important role in regulating the lipid biosynthesis and metabolic pathways. Overexpression of the soybean TF *GmDof4* significantly enhances the lipid content of *Chlorella ellipsoidea* (Zhang et al., 2014). Kang et al. reported that *Wrinkled1*, a TF of *Arabidopsis*, enhanced lipid production in the microalgae *Nannochloropsis salina* (Kang et al., 2017).

*LEC1* is a central regulator that controls many aspects of seed development, including the maturation phase during which seeds accumulate storage macromolecules and embryos acquire the ability to withstand desiccation in *Arabidopsis* (West et al., 1994). The induced overexpression of *LEC1* can affect *ABI3*, *FUS3*, *WRINKLED1* and other TFs and improve the overall level of fatty acid synthesis-related gene expression (Mu et al., 2008). Shen et al. (2010) found that the overexpression of corn *ZwLEC1* gene under embryo-specific weak promoter *EPA1* could significantly increase the oil content of transgenic maize, but plant leaves are reduced to approximately half of the leaves of the wild type. *C. ellipsoidea* is a unicellular eukaryotic organism that has no differentiation of tissues and may be a good receptor of *LEC1* overexpression without lethal or harmful effects to the host cell.

In this study, we investigated the feasibility and the mechanism for improving the lipid content of *C. ellipsoidea* by the overexpression of *AtLEC1*. The results indicated that the lipid content of transgenic *C. ellipsoidea* strains was significantly increased under mixotrophic and autotrophic culture conditions, but the growth rate of the strains was not affected. In addition, RNA-seq data showed that *AtLEC1* significantly regulated 924 genes of *C. ellipsoidea*, and we found the regulation mechanism of *AtLEC1* in *C. ellipsoidea* to have some differences compared with the regulation mechanism in *Arabidopsis*. Our study provided a new route for engineering microalgae to increase the lipid content and help to elucidate the mechanism of lipid accumulation in *C. ellipsoidea* regulated by *LEC1* from a higher plant.

## Materials and Methods

### Strain and Culture Conditions

The *C. ellipsoidea* strains used in this study were grown in Endo medium (Appleyard, 1954) for the mixotrophic culture and in KNOP medium (McLEOD, 1958) for the autotrophic culture in a rotary shaker (DZ-900, Zhongkepusen Co., Ltd., Beijing, China), 200 rpm at 25°C under illumination (100 μmol photons/m^2^/s).

### *AtLEC1* Expression Vector Construction and Transformation of *C. ellipsoidea*

**FIGURE 1:**
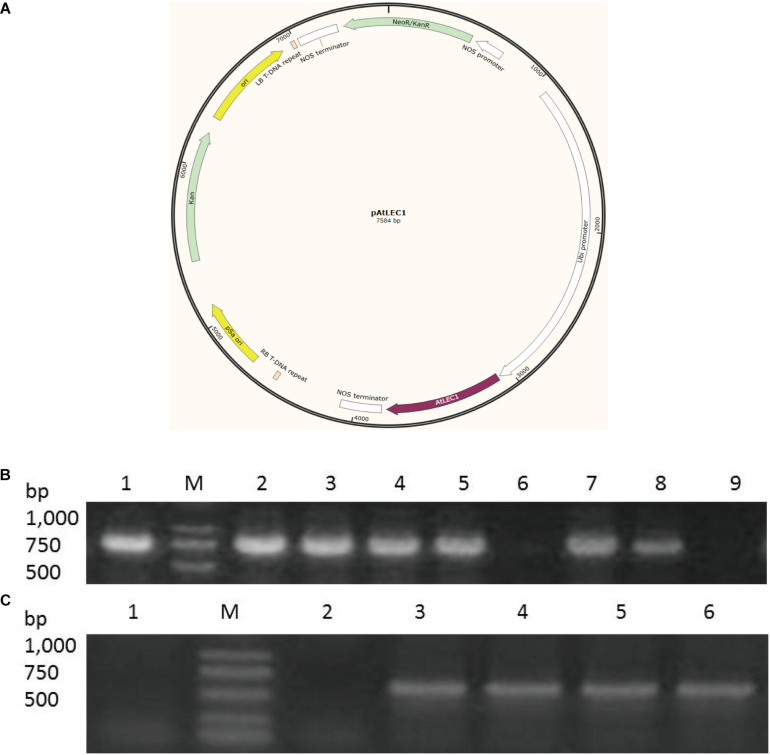
*AtLEC1* transformation vector and detection of *AtLEC1* transgenic strains. **(A)** A schematic map of the *AtLEC1* plasmid. **(B)** PCR analysis of WT, CK, and *AtLEC1* transgenic lines. M: Marker; 1: *pAtLEC1* vector; 2-5: LEC1-1, LEC1-2, LEC1-3, LEC1-4; 6: CK; 7-8: LEC1-5, LEC1-6; 9: WT. **(C)** Detection of the expression of *AtLEC1* in transgenic lines by RT-PCR. M: Marker; 1: WT; 2: CK; 3-6: LEC-1, LEC-2, LEC-3, LEC-4.

The cDNA of *AtLEC1* was generously provided by Prof. Jianru Zuo (Institute of Genetics and Developmental Biology, Chinese Academy of Sciences). The *AtLEC1* cDNA was cloned into a T-vector (pEASY-Blunt Cloning Vector, TransGen Biotech. Ltd., Beijing, China), and later inserted at *Spe*I and *Not*I sites of pGreen0029 driven by UBI promoter from maize, which was named pGreen0029-Ubi-AtLEC1-Nos (*pAtLEC1*) (Figure 1A). *C. ellipsoidea* was transformed using plasmid *pAtLEC1* according to the previously described method (Bai et al., 2013). Briefly, strains were cultured to the logarithmic phase in Endo medium, mixed with 0.2 M mannitol and 0.2 M sorbitol and kept on ice for 1 h. The resuspended strains were mixed with electroporation buffer (0.08 M KCl, 0.005 M CaCl_2_, 0.01 M HEPES, 0.2 M mannitol, and 0.2 M sorbitol), a final concentration of 20 μg/mL *pAtLEC1* plasmid, a final concentration of 10 μg/mL plasmid pSoup, and 25 μg/mL salmon sperm DNA. The strains were transformed with a Baekon 2000 (Baekon Co., CA, United States) electroporation device. After electroporation, the strains were screened using SE agar selection medium containing 30 mg/L G418. The selected individual strains were subcultured in SE liquid medium containing 15 mg/L G418.

### Characterization of Transgenic Strains With PCR

Genomic DNA isolation, total RNA isolation, PCR amplification, and cDNA synthesis were performed as previously described (Zhang et al., 2014). All relevant primer sequences used for PCR and RT-PCR are listed in Supplementary Table 1. Identification of transgenic strains was carried out using a pair of primers, P1 and P2. The reaction conditions were as follows: denaturation at 95°C for 10 min, 30 cycles of 94°C for 30 s, 55°C for 30 s, and 72°C for 2 min followed by final extension at 72°C for 10 min. Total RNA was isolated using an RNA extraction kit (Takara). The reverse transcriptome product was diluted by a factor of 10 for RT-PCR amplification, in which total 20 μL reaction mixtures contained 10 μL 2 × M5 HiPer plus Taq HiFi PCR Mix (Mei5 Biotechnology), 1 μL primer P3, 1 μL primer P4, 1 μL template, and 7 μL ddH_2_O. The reaction conditions were as follows: denaturation at 94°C for 5 min, 28 cycles of 94°C for 1 min, 55°C for 1 min, and 72°C for 20 s, and a final extension step of 72°C for 5 min. RT-PCR products were analyzed by electrophoresis on 1% agarose gel. For the verification of the selected gene expression level, qRT-PCR analyses were performed on a LightCycler^®^ 480. The 20-μL reaction mixtures contained 10 μL EvaGreen 2 × qPCR MasterMix (abmGood.com), 1 μL of each primer (10 μM), 1 μL template, and 7 μL ddH_2_O. The reaction conditions were as follows: 1 cycle 95°C for 10 min, 40 cycles of 95°C for 10 s, and 60°C for 30 s. To normalize the amount of transcripts in each sample, the relative abundance of 18S rRNA was determined and used as an internal standard control. The gene expression value was the difference (Ct) between the target gene and the reference gene.

### Biomass and Everyday Growth Rate Analysis

The biomass of *AtLEC1* transgenic strains and the WT were analyzed under mixotrophic and autotrophic culture conditions at 25°C and illumination (100 μmol photons/m^2^/s) in Endo medium and KNOP medium, respectively. The *C. ellipsoidea* biomass concentration (w/v) was equivalent to a specific value of the strain dry weight (DW) that was determined by OD 540 according to the following empirical formula:

(1) DW⁢(g/L)=(OD540+ 0.0097)/0.4165

The everyday growth rate (EGR) was calculated according to the equation (White et al., 1991):

(2) EGR=(X2-X1)/X1

X1 was the biomass concentration on the initial day; X2 was the biomass concentration on the next day.

### Measurement of the Soluble Proteins, Carbohydrate, Lipid Content, and the Fatty Acid Composition

To measure the daily growth rate (DGR) and biomass of the *AtLEC1* transgenic and the WT strains, we collected cultured algae after the 1st day (1D), 5th day (5D), and 9th day (9D) under mixotrophic culture conditions and the 5th day (5D), 10th day (10D), and 15th day (15D) under autotrophic culture conditions. Each sample had three biological replicates, and the freeze-dried biomass was collected to measure the soluble proteins, carbohydrate, lipid content, and fatty acid composition. The carbohydrate content was analyzed based on the procedure published by Miao and Wu (2004). Proteins were extracted following the procedure of Rausch (1981) and were quantified using the Bradford method (Bradford, 1976). Lipid extraction was performed by the Soxhlet method that was similar to the procedures reported by Folch et al. (1957). The fatty acid compositions were qualitatively and quantitatively determined using a TurboMass Gas Chromatograph Mass Spectrometer (PerkinElmer, MA, United States) with a capillary column (BPX-70, 30 m × 0.25 mm × 0.25 μm) using the method as previously described (Song et al., 2010). The Nile red staining followed a previous method (Greenspan et al., 1985), which was used to visualize the intracellular lipid bodies as indicators of TAG formation.

### Photosynthetic Pigment Content Measurement

Photosynthetic pigment content was measured according to the previous method (Fargasová and Molnárová, 2010). Briefly, 0.02 g of a freeze-dried *Chlorella* powder sample was mixed with 4 mL of 95% (v/v) ethanol in an airtight tube and agitated at room temperature overnight in the dark until the color turned to white. After centrifugation at 5,000 rpm for 5 min, the ethanol phase was removed, and more 95% (v/v) ethanol was added to a volume of 25 mL. Next, the sample was measured with a spectrophotometer at wave lengths of 665 nm (A665), 649 nm (A649), and 470 nm (A470). The 95% ethanol was used as a blank control. The amount of chlorophyll a (Ca) was calculated as Ca = 13.95 × A665 − 6.88 × A649; chlorophyll b (Cb) as Cb = 24.96 × A649 − 7.32 × A665; and carotenoids (Cc) as Cc = (1000 × A470 − 2.05 × Ca − 114.8 × Cb)/245. The total content of chlorophyll per fresh weight was calculated as C = 2 × (Ca + Cb + Cc)/W.

### Illumina-Based RNA-Seq Analysis

For the gene expression analysis by RNA-seq, the transgenic *AtLEC1-1* strain and the WT strain were collected on the 5th day of cultivation under mixotrophic conditions. Three independent biological replicates were used for the data analysis. The cDNA library was sequenced on an SE flow cell using Illumina Genome Analyzer IIx (Illumina, San Diego, CA, United States). Finally, 8.08 Gb clean data (total) with more than 90.92% of Q30 were generated from two GAIIx single-end lanes. Using SOAPdenovo with the parameters “-K31–d3–R,” 775,293 contigs with an N50 contig size of 2,072 bp were obtained (Li et al., 2009). To detect the differentially expressed genes (DEGs), we first mapped the short reads to the reference genes using the Burrows Wheeler Alignment tool (BWA) program with default parameters. For the validation and annotation of the assembled contigs, a sequence similarity search was conducted against a non-redundant protein database using the BLASTx algorithm with an E-value threshold of 10^–3^. The results demonstrated that of 13,566 contigs, 7,559 (55.72%) showed significant similarity to known proteins in the non-redundant (Nr) database. Contigs with a similarity greater than the threshold were annotated using GO, the molecular function, biological process, and cellular component ontologies^1^ by the Blast2GO program (Conesa et al., 2005). The RNA-seq data (PRJCA003770) were available in the BIG Data Center^2^.

### Analysis of Sequence Similarity

To detect protein sequence similarities of LEC1 among different species, a total of 13 homologous genes of *AtLEC1* were selected from *Glycine max*, *Brassica napus*, *Micromonas pusilla*, *Micromonas commode*, *Coccomyxa subellipsoidea*, *Ostreococcus lucimarinus*, *C. reinhardtii*, *Volvox carteri*, *Dunaliella salina*, *Saccharomyces cerevisiae*, *Homo sapiens*, and *Mus musculus*, and the sequence similarity analysis was subsequently performed through software DNAstar v7.1.0. Phylogenetic tree was inferred using the neighbor-joining method and the bootstrap consensus tree inferred from 1,000 replicates in MEGA v7.0 (Kumar et al., 2016).

### Statistical Analysis

*P*-values (means ± SD) were calculated with Student’s *t*-test (two-tailed) by Microsoft Excel. ^∗^Significance at *p* < 0.05 and ^∗∗^ significance at *p* < 0.01 were used for the comparison with the control based on Student’s *t*-test. The experimental replicates, sample size, and significance level of *p*-values are described in the figure legends.

## Results

### *AtLEC1* Expression in *C. ellipsoidea* Does Not Affect Growth

**FIGURE 2:**
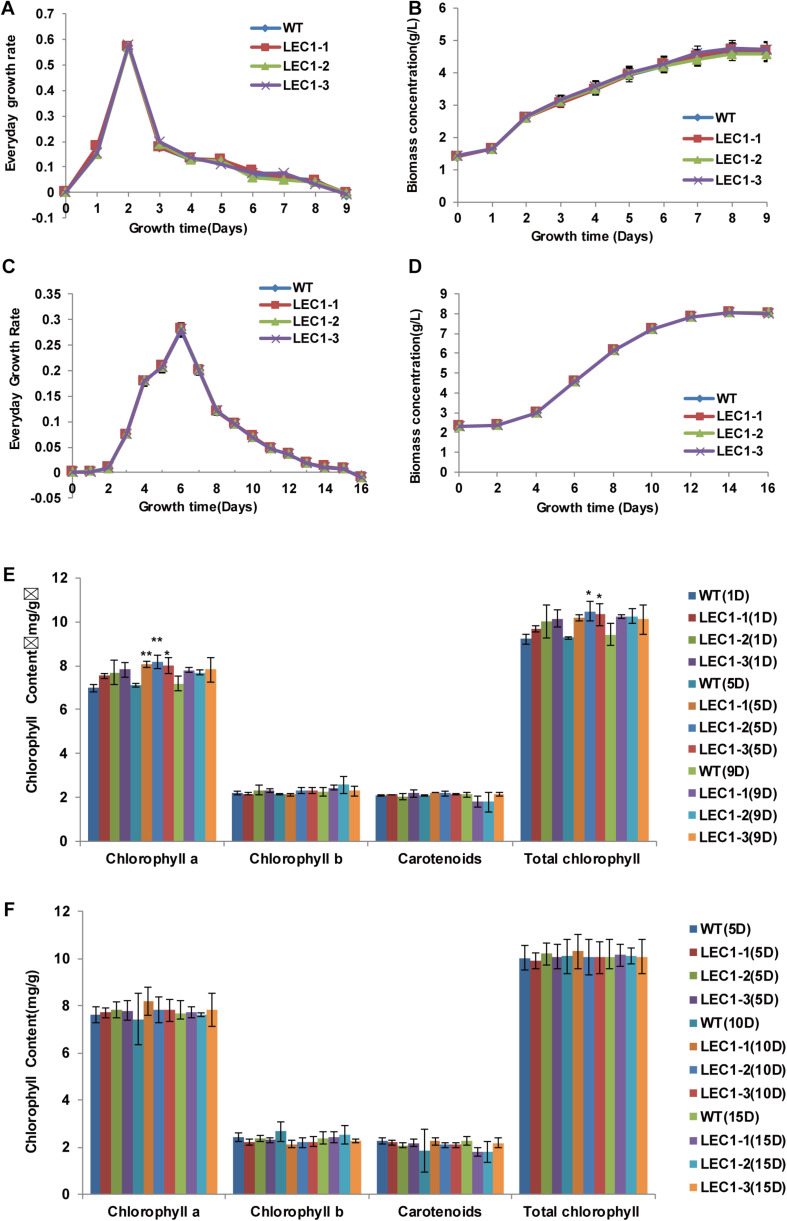
Growth characterization of *AtLEC1* transgenic *Chlorella ellipsoidea*. **(A)** Every growth rate (EGR) of *AtLEC1* transgenic strains under mixotrophic culture conditions for 9 days. **(B)** The biomass of *AtLEC1* transgenic strains under mixotrophic culture conditions. **(C)** The EGR of *AtLEC1* transgenic strains under autotrophic culture conditions for 16 days. **(D)** The biomass of *AtLEC1* transgenic strains under autotrophic culture conditions. **(E)** The chlorophyll content of *AtLEC1* transgenic strains under mixotrophic culture conditions. **(F)** The chlorophyll content of *AtLEC1* transgenic strains under autotrophic culture conditions. WT, wild type; LEC1-1, LEC1-2, LEC1-3, three *AtLEC1* transgenic strains. The values are the means of three biological replicates. Asterisks indicate statistically significant differences, Student’s *t*-test: *p < 0.05, **p < 0.01 compared with WT under the same conditions.

In our study, we transferred an *Arabidopsis* gene *AtLEC1* into *C. ellipsoidea* using the *AtLEC1* expression vector *pAtLEC1* according to a previous method (Bai et al., 2013). A schematic map of the *AtLEC1* plasmid (Figure 1A), PCR analysis of *AtLEC1* transgenic strains and detection of the expression of *AtLEC1* in transgenic strains by RT-PCR are presented in Figures 1B,C. The primers used in our study are listed in Supplementary Table 1. The *AtLEC1* transgenic and wild-type (WT) *C. ellipsoidea* strains were cultured according to a previously described method (Zhang et al., 2014). To detect the effect of *AtLEC1* on *C. ellipsoidea* growth, the DGR and the biomass concentration of transgenic and WT were measured under mixotrophic and autotrophic conditions. The growth curves of *AtLEC1* transgenic strains showed no significant difference compared with the growth curves of WT under mixotrophic culture conditions (Figures 2A,B) and autotrophic culture conditions (Figures 2C,D). Photosynthetic pigment content was an important growth index in *C. ellipsoidea*. We collected the strains on the 1st day, 5th day, and 9th day of cultivation to measure the chlorophyll content, including chlorophyll a, chlorophyll b, carotenoids, and total chlorophyll content under mixotrophic culture conditions. The result indicated that the total photosynthetic pigment content of WT strains ranged between 9.22 and 9.42 mg/g, and transgenic strain LEC1-1 was 9.69–10.25 mg/g, LEC1-2 was 10.02–10.47 mg/g, and LEC1-3 was 10.10–10.32 mg/g. Except for the chlorophyll a higher in transgenic strains than WT, there were no significant differences between them (Figure 2E). In addition, there was no significant difference between *AtLEC1* transgenic strains and WT cultured on the 5th day, 10th day, and 15th day under autotrophic culture conditions (Figure 2F). In other words, the *AtLEC1* transformation did not affect the growth and photosynthesis in *C. ellipsoidea*, which established a foundation for further research.

### Expression of *AtLEC1* Increases the Lipid Content in *C. ellipsoidea*

**FIGURE 3:**
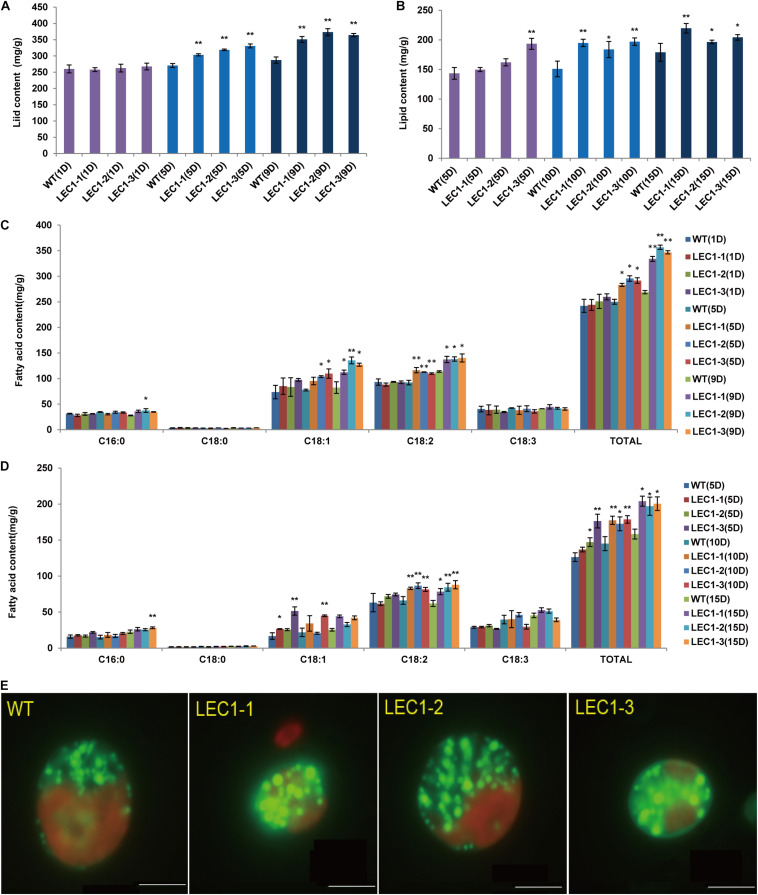
Effect of *AtLEC1* on *Chlorella* fatty acid composition and lipid content. **(A)** The lipid content in *AtLEC1* transgenic strains under mixotrophic culture conditions. **(B)** The lipid content in *AtLEC1* transgenic strains under autotrophic culture conditions. **(C)** The fatty acid composition and total fatty acids in *AtLEC1* transgenic strains under mixotrophic culture conditions. **(D)** The fatty acid composition and total fatty acid content in *AtLEC1* transgenic strains under autotrophic culture conditions. **(E)** Observation and determination of oil droplets in *C. ellipsoidea* and WT by Nile red staining. WT, wild type; LEC-1, LEC-2, LEC-3, three *AtLEC1* transgenic strains; Bars = 5 μm. The values are the means of three biological replicates. Asterisks indicate statistically significant differences, Student’s *t*-test: *p < 0.05, **p < 0.01 compared with WT under the same conditions. 1D, 5D, 9D, 10D, and 15D were the 1st, 5th, 9th, 10th, and 15th days, respectively.

**FIGURE 4:**
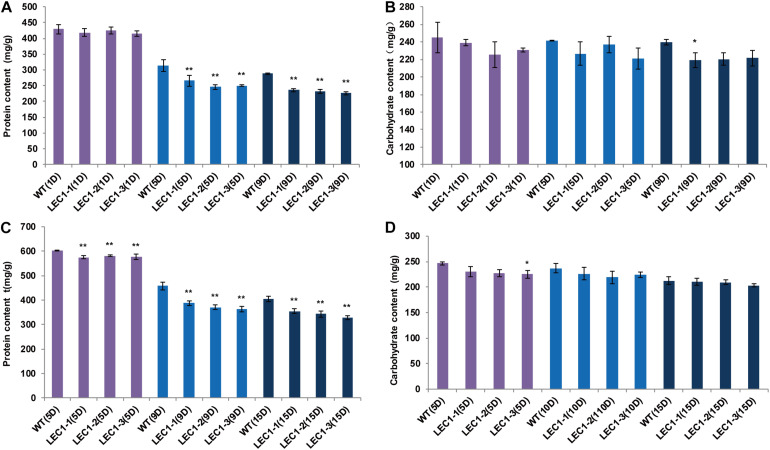
Effect of *AtLEC1* on protein content and carbohydrate content. **(A)** The protein content in *AtLEC1* transgenic strains under mixotrophic culture conditions. **(B)** The carbohydrate content in *AtLEC1* transgenic strains under mixotrophic culture conditions. **(C)** The protein content under autotrophic culture conditions. **(D)** The carbohydrate content in *AtLEC1* transgenic strains under autotrophic culture conditions. 1D, 5D, 9D, 10D, and 15D were the 1st, 5th, 9th, 10th, and 15th days, respectively.

Compared with WT, the lipid productivity of transgenic strains was significantly higher than the lipid productivity of WT under mixotrophic and autotrophic culture conditions. Under mixotrophic culture conditions (Figure 3A), on the 5th day, the lipid content of WT was 270.64 mg/g, while the lipid content of transgenic strains LEC1-1, LEC1-2, and LEC1-3 was increased by 12.14–22.28%, reaching 303.51–330.95 mg/g. On the 9th day, the lipid content was 287.33 mg/g, while the lipid content of transgenic strains LEC1-1, LEC1-2, and LEC1-3 increased by 22.14–29.91%. Under autotrophic culture conditions (Figure 3B), on the 10th day, the lipid content of the WT was 150.96 mg/g, while the lipid content of the transgenic strain was increased by 21.69–30.45%. On the 15th day, the lipid content of transgenic strains was increased by 9.28–22.77%. Gas chromatography/mass spectrometry (GC/MS) analysis indicated that the main types of fatty acids of *AtLEC1* transgenic strains and WT were not changed, but the content of total fatty acids C18:1 (oleic acid) and C18:2 (linoleic acid) increased significantly in the transgenic strains under both mixotrophic and autotrophic culture conditions (Figures 3C,D). Under mixotrophic culture conditions, on the 5th day, the C18:1, C18:2 and total fatty acid content of the transgenic strains increased by 22.70–41.25, 19.07–26.14, and 13.19–18.16%, respectively. On the 9th day, the C18:1, C18:2 and total fatty acid content of the transgenic strains increased by 36.40–64.58, 20.67–23.25, and 24.20–32.65%, respectively (Figure 3A). Under autotrophic culture conditions, on the 5th, 10th, and 15th days of cultivation, the total fatty acid content of the transgenic strains increased by 8.29–39.61, 18.88–23.13, and 24.40-28.87%, respectively (Figure 3B). The lipid increase in transgenic strains could also be clearly observed by Nile red staining (Figure 3E). Oil droplet fluorescence was measured on a Varian 96-well plate spectrofluorometer, and the results showed that transgenic strains can accumulate more oil droplets than WT strains. At the same time, the carbohydrate content of the three transgenic strains was not significantly different (Figures 4B,D), while the protein content was significantly decreased (Figures 4A,C) compared to the protein content of the WT. In brief, under mixotrophic culture on the 9th day, the protein content in WT was 288.53 mg/g, the protein content in LEC1-1 was decreased by 18.23%, reaching 235.93 mg/g, the protein content in LEC1-2 was decreased by 19.67%, reaching 231.77 mg/g, and the protein content in LEC1-3 was decreased by 21.44%, reaching 226.66 mg/g. Under autotrophic culture for 15 days, the protein content in WT was 404.13 mg/g, in LEC1-1 was decreased by 12.28% reaching 354.50 mg/g, in LEC1-2 was decreased by 14.99% reaching 343.53 mg/g, and in LEC1-3 was decreased by 18.66% reaching 328.71 mg/g. These results demonstrated that the overexpression of *AtLEC1* significantly increased oil production and decreased the protein content in *C. ellipsoidea*.

### Transcriptome Analysis of *AtLEC1* Strains

**FIGURE 5:**
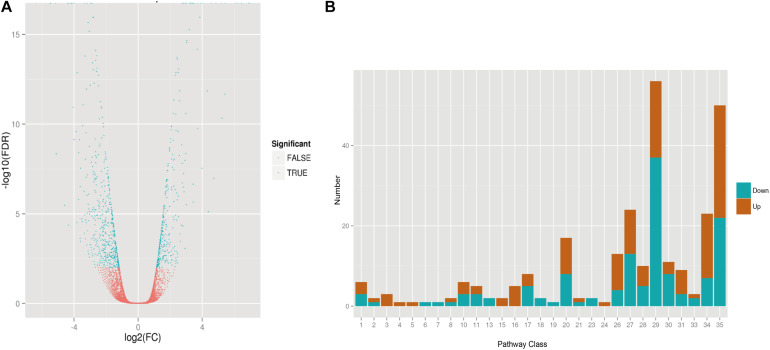
RNA-seq in transgenic *AtLEC1-1 Chlorella* under mixotrophic culture conditions. **(A)** Volcano plot showing the differential expression gene analysis of transgenic *AtLEC1-1* and wild-type strains. **(B)** Differential expression analysis of *AtLEC1-1* strains involved in metabolic regulation. Notes: 1. Photosynthesis; 2. Major carbohydrate metabolism; 3. Minor CHO metabolism; 4. Glycolysis; 5. Fermentation; 6. Gluconeogenese/glyoxylate cycle; 7. Oxidative Pentose Phosphate; 8. TCA/org conversion; 10. Cell wall synthesis; 11. Lipid synthesis; 13. Amino acid handling factor; 15. Metal stress; 16. Secondary metabolism; 17. Hormone synthesis; 18. Cometabolism; 19. Pyrrole metabolism; 20. Coercion; 21. Redox regulation; 23. Nucleotide synthesis; 24. Biodegradation of xenobiotics; 26. Others; 27. RNA; 28. DNA; 29. Protein; 30. Signal; 31. Cells; 33. Developmental diversity; 34. Carrier protein; 35. Not classified.

Transcriptome analyses for transgenic *AtLEC1-1* and WT strains were obtained using the Illumina GAIIx platform. The RNA sample was collected at the stage of mixotrophic culture conditions for 5 days. After the quality control, 38,967 unigenes with an average length of 791 bp were obtained, 7,788 unigenes of which can be annotated by public databases such as COG, GO, KEGG, Swiss-Prot, NR, and pfam. We found expression of 10,823 genes was changed, including 5,413 upregulated genes and 5,410 downregulated genes (Supplementary Table 2). To explore the functional information of unigene transcription, MapMan v3.5^3^ was used to classify the metabolic pathways. According to the criteria to determine differential expression of genes [false discovery rate (FDR) ≤ 0.01] (Ness et al., 2011), 924 DEGs were identified, including 360 significantly upregulated genes and 564 significantly downregulated genes (Figure 5A), and 471 DEGs were annotated by different public databases (Supplementary Table 2). Compared with the transcriptome data of *Arabidopsis* with overexpression of *AtLEC1*, 269 of 924 DEGs in *C. ellipsoidea* showing protein sequence similarity with 245 genes from *Arabidopsis* were classified into 28 known pathways (Figure 5B and Supplementary Figure 1). Compared with WT, DEGs relative to five pathways were significantly upregulated in *AtLEC1* strains, including the minor CHO metabolism, the glycolysis, the fermentation, the metal stress and the secondary metabolism (Supplementary Figure 1). In contrast, DEGs relative to six pathways were significantly downregulated, including the gluconeogenesis/glyoxylate cycle, the oxidative pentose phosphate, the amino acid metabolism, the cofactor metabolism, the tetrapyrrole synthesis, and the nucleotide metabolism (Supplementary Figure 1). The transcriptome indicated that some of these genes were significantly changed in protein, RNA metabolism and transporter in the *AtLEC1* transgenic strains (Supplementary Table 3). Therefore, these genes may be involved in the regulation of lipid accumulation in *AtLEC1* transgenic *Chlorella*.

### Verification of the Regulatory Function of *AtLEC1* by qRT-PCR

**FIGURE 6:**
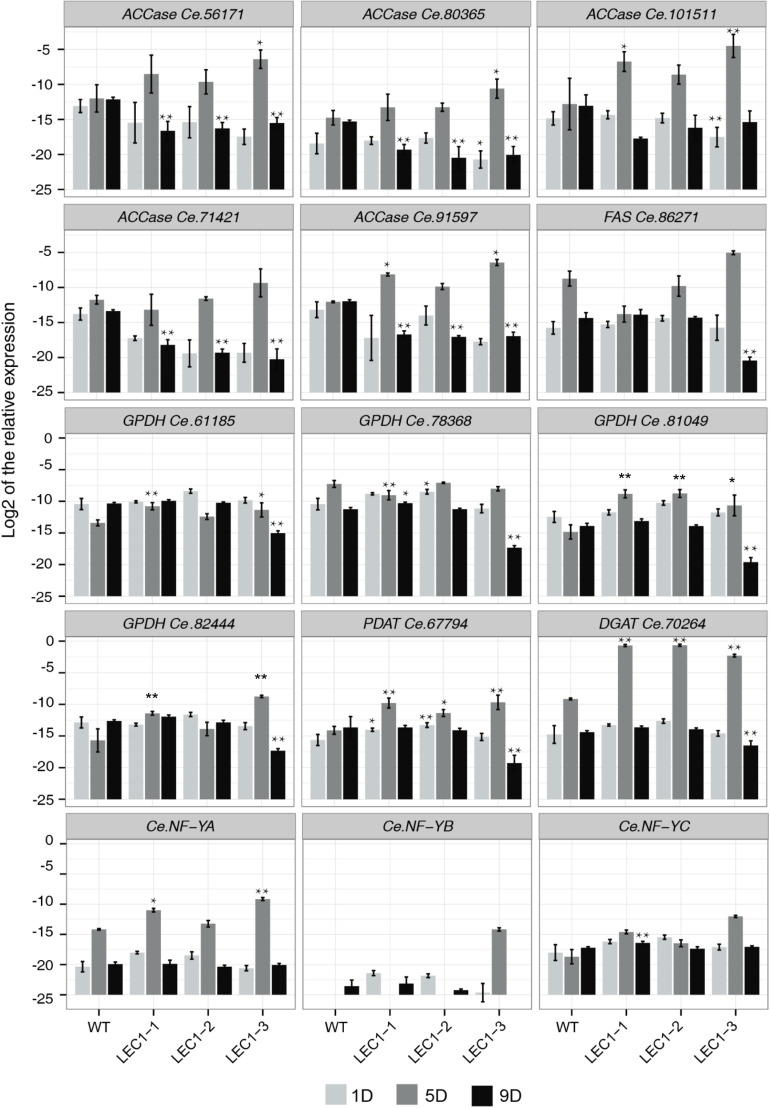
Expression of lipid accumulation-related genes in *AtLEC1* transgenic strains. WT, wild type; LEC-1, LEC-2, LEC-3, three *AtLEC1* transgenic strains; 1D, 5D, and 9D were sampled on the 1st, 5th, and 9th days under mixotrophic culture conditions. The relative abundance of 18S rRNA was used as an internal standard control. The values are the difference (Ct) between the target gene and the reference gene. The values are the means of three biological replicates. Asterisks indicate statistically significant differences, Student’s *t*-test: *p < 0.05, **p < 0.01 compared with WT under the same conditions.

The relative expression level of 15 significantly regulated genes associated with lipid and fatty acid metabolism were analyzed by qRT-PCR (Figure 6). The WT and transgenic LEC1-1, LEC1-2, and LEC1-3 strains were collected on the 1st day, 5th day, and 9th day of cultivation under mixotrophic conditions. Three independent transgenic strains were analyzed. The results indicated that the expression levels were the highest on the 5th day (logarithmic stage) and decreased on the 9th day (plateau stage). The results (Figures 2B, 3A) suggested that the accumulation of biomass and lipid content reached its maximum value on the 9th day, indicating that the gene expression was earlier than the lipid accumulation. Therefore, the strain growth on the 5th day may be a vital period for lipid accumulation in *C. ellipsoidea*. Furthermore, we observed that in transgenic *AtLEC1* strains on the 5th day, the expression levels of some genes related to lipid synthesis were higher compared with the genes in WT, such as *ACC* (*Ce.101511* and *Ce.91597*), *GPDH* (*Ce.61185*, *Ce.81049*, and *Ce.82444*), *PDAT* (*Ce.67794*), *DGAT* (*Ce.70246*), and *NF-Y* (*Ce.NF-YA*) (Figure 6). Interestingly, the expression level of *DGAT1* (*Ce.70246*) in transgenic strains was 2^6.85^–2^8.5^ times higher than the expression level in WT. *DGAT* was the rate-limiting enzyme of TAG synthesis in the Kennedy pathway (Lehner and Kuksis, 1996). In addition, we found there were no significant differences for partial genes of *ACCase*, *FAS*, and *GPDH*, such as *ACCase* (*Ce.56171*, *Ce.80365* and *Ce.71421*), *FAS* (*Ce.86271*), and *GPDH* (*Ce.78368*), in transgenic *AtLEC1-1* and WT, which may function diversely. These results suggested that *AtLEC1* in *Chlorella* could increase the expression level of such genes as *ACC* and *GPDH*, especially *DGAT*, thereby helping to explain the increasing lipid accumulation in *Chlorella*.

### *AtLEC1* Regulates the Protein and Carbohydrate Metabolic Networks

**FIGURE 7:**
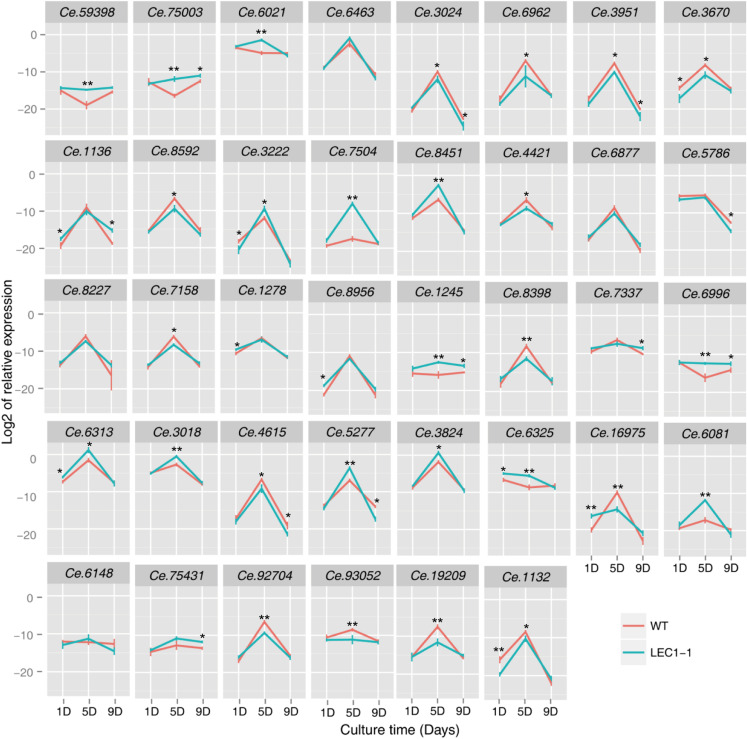
Expression of protein and carbohydrate metabolism-related genes in *AtLEC1* transgenic strains. WT, wild type; *LEC-1*, *AtLEC1* transgenic strain; 1D, 5D, and 9D were sampled on the 1st, 5th, and 9th days under mixotrophic culture conditions. The relative abundance of 18S rRNA was used as an internal standard control. The values are the difference (Ct) between the target gene and the reference gene. The values are the means of three biological replicates. Asterisks indicate statistically significant differences, Student’s *t*-test: *p < 0.05, **p < 0.01 compared with WT under the same conditions.

To investigate whether the protein and carbohydrate were involved in *AtLEC1* metabolism regulation, WT and transgenic LEC1-1, LEC1-2, and LEC1-3 strains were cultured on the 1st day, 5th day, and 9th day under mixotrophic conditions. The candidate genes related to protein and carbohydrate metabolism were selected (Supplementary Table 4), and the expression level of these genes was detected by qRT-PCR to verify the regulation function of *AtLEC1* (Figure 7). On the 5th day of culture, the expression level of protein biosynthesis-related genes, such as *Ce.59398* (nitrogen assimilation regulatory), *Ce.75003* (nitrite transporter), *Ce.6021* (ribosomal protein 60S subunit L23), *Ce.6463* (TCP-1/cpn60 chaperonin family protein), *Ce.3024* (TCP-1/cpn60 chaperonin family protein), and *Ce.6962* (germin-like protein), were downregulated. The expression level of storage protein-related genes in WT, such as *Ce.6962* and *Ce.3951* (RmlC-like cupin superfamily protein), was approximately 16- and 4-fold, similar to the expression level of storage protein-related genes in LEC1-1, respectively. These results may explain why the protein accumulation significantly decreased in the *AtLEC1* transgenic strain compared with the protein accumulation in WT. Among six selected genes that were related to carbohydrate metabolism (*Ce.3222*, *Ce.7504*, *Ce.8451*, *Ce.4421*, *Ce.6877*, and *Ce.5786*), the expression levels of *Ce.3222* and *Ce.7504* genes were significantly increased in *AtLEC1* transgenic strains on the 5th day. *Ce.3222* and *Ce.7504* may be involved in Rubisco function in the dark reaction of photosynthesis, and their increased expression could increase the raw materials for *de novo* synthesis of fatty acids.

Among eight selected genes that were related to signal transduction (*Ce.8227*, *Ce.7158*, *Ce.1278*, *Ce.8956*, *Ce.1245*, *Ce.8398*, *Ce.7337*, and *Ce.6996*), the expression level of *Ce.1245* and *Ce.6996* was upregulated in *AtLEC1* transgenic strains on the 5th day of culture. Among 7 selected genes that were related to material transport (*Ce.6313*, *Ce.3018*, *Ce.4615*, *Ce.5277*, *Ce.3824*, *Ce.6325*, and *Ce.16975*), the expression level of five genes was downregulated in *AtLEC1* transgenic strains, except for *Ce.4615* and *Ce.16975*. The expression levels of the genes encoding short-chain fatty acid dehydroreductase, *Ce.19209* and *Ce.1132*, were downregulated in *AtLEC1* transgenic strains.

## Discussion

### *AtLEC1* Increased the Lipid Content but Did Not Affect the Growth in *C. ellipsoidea*

With the development of biotechnology and molecular biology, multiple approaches have provided insight into the mechanisms of lipid synthesis and accumulation in microalgae. Based on the transcriptome and lipidome of *C. reinhardtii* under heat stress, a phospholipase A2 homolog and the DAG acyltransferase gene *DGTT1* were identified (Légeret et al., 2016). Cecchin et al. (2020) sequenced the nuclear and organelle genomes of *C. vulgaris 211/11P* by combining next-generation sequencing and optical mapping of isolated DNA molecules and identified 10,724 nuclear genes, 121 chloroplast genes and 48 mitochondrial genes. *LEC1* is an important TF, and its function in microalgae has not been elucidated. In our study, the total fatty acid content and total lipid content in *AtLEC1* transgenic *C. ellipsoidea* strains were significantly increased by 24.2–32.65 and 22.14–29.91% under mixotrophic culture conditions, respectively. Under autotrophic conditions, the total fatty acid content and total lipid content were significantly increased by 24.4–28.87 and 21.69–30.45%, respectively. Notably, the overexpression of *AtLEC1* did not affect the growth rate of strains, but the protein content significantly decreased. In higher plants, *LEC1* is expressed primarily in embryonic tissues and plays an important biological function in controlling late embryonic development and cotyledon formation (Kwong et al., 2003; Wang and Perry, 2013). Loss-of-function *Lec1* mutations cause phenotypically abnormal embryos (Meinke, 1992), defects in storage protein and lipid accumulation (Santos-Mendoza et al., 2008), and etiolation-related phenotypes in early seedlings in *Arabidopsis* (Huang et al., 2015). The overexpression of *BnaLEC1* under a seed-specific promoter (*Napin A*) from *B. napus* caused the *Arabidopsis* transgenic plants to be abnormal after germination with complete death or sterility. However, when the promotor activity is only 18% of the original, plant growth and propagation are normal with a remarkable improvement in lipid content (Tan et al., 2011). Our results suggested that *AtLEC1* can improve microalgae oil productivity and that it has advantages for improving *Chlorella* lipid content over that of higher plants, which can eliminate the unfavorable characteristics caused by the different tissue and organ differentiation of higher plants.

### *AtLEC1* Regulated the Gene Expression of *C. ellipsoidea*

*LEC1* served as a key regulator to coordinate the expression of fatty acid biosynthetic genes. In our study, key genes related to lipid synthesis were identified, including *ACCase* (*Ce.101511* and *Ce.91597*), *GPDH* (*Ce.61185, Ce.81049*, and *Ce.82444*), *PDAT1* (*Ce.67794*), and *DGAT1* (*Ce.70246*). Their expression levels were higher in the transgenic strains than in the WT. *ACCase* activity is directly related to fatty acid accumulation (Leyva et al., 2014). *DGAT* is the rate-limiting enzyme of TAG synthesis in the Kennedy pathway (Lehner and Kuksis, 1996). Our results showed that *AtLEC1* could enhance the expression of lipid biosynthesis-related genes to improve lipid content in *C. ellipsoidea*. *LEC1* encodes a nuclear factor YB (NF-YB) subunit of NF-Y (or the CCAAT box–binding factor HAP3), which is a heterotrimer consisting of three subunits (NF-YA, NF-YB, and NF-YC) and is highly conserved in all eukaryotic organisms (Edwards et al., 1998; Lotan et al., 1998; Mu et al., 2013). Although the homolog of AtLEC1 was not detected in our RNA-seq, three members of the NF-Y gene family, *CeNF-YA*, *CeNF-YB*, and *CeNF-YC*, were found. Only the expression of *CeNF-YA* was enhanced in the transgenic cells. The results suggested that the expression of one of the NF-Y members can be affected by *AtLEC1* and may contribute to the increase in lipid accumulation.

Several studies have indicated that *LEC1* is directly involved in regulating photosynthesis and chloroplast function during seed development in higher plants (Willmann et al., 2011; Allorent et al., 2015). *LEC1* controls distinct gene sets at different developmental stages, *LEC1* binding alone does not appear to be sufficient to regulate gene expression, and *LEC1* function is partially dependent on *ABSCISIC ACID INSENSITIVE3 (ABI3)*, *FUSCA3 (FUS3)*, and *WRINKLED1 (WRI1)* in the regulation of fatty acid biosynthesis in higher plants (Mu et al., 2008; Pelletier et al., 2017; Jo et al., 2020). However, there were no members of the ABI3VP1/B3 family in our transcriptomic database; therefore, the homologous genes of *FUS3*, *ABI3* or *LEC2* were not found. *WRI1* belongs to *AP2-EREBP/ERF*, which plays an important role in fatty acid biosynthesis in higher plants (Focks and Benning, 1998). In our study, 10 TF genes (*AP2-EREBP/ERF* family) were selected, namely, *Ce.7138*, *Ce.4614*, *Ce.1006*, *Ce.3182*, *Ce.6245*, *Ce.5515*, *Ce.5831*, *Ce.2945*, *Ce.11419*, and *Ce.2372*, for their expression detection in WT, and *AtLEC1* transgenic *Chlorella* grown under mixotrophic culture for 1, 5, and 9 days was analyzed by qRT-PCR. Surprisingly, the expression of these genes was not detectable (data not shown). The differential regulatory patterns due to the great genomic diversity between higher plants and unicellular green algae need to be studied further.

### Difference Between the Genes Regulated by *AtLEC1* in *Arabidopsis* and in *Chlorella*

To analyze the differences in the regulatory network affected by *AtLEC1* between *Chlorella* and higher plants, the transcriptomes of *AtLEC1 Chlorella* and *AtLEC1* transgenic *Arabidopsis* (GSE12137) were analyzed. As in previous studies, in *AtLEC1* transgenic *Arabidopsis*, approximately 425 genes were significantly upregulated and 262 genes were significantly downregulated in transgenic seedlings when *AtLEC1* was induced by estradiol, and over 58% of known enzyme-coding genes were upregulated in the plastidial fatty acid synthetic pathway (Mu et al., 2008). In our study, according to the annotation, 220 regulated *Chlorella* genes were annotated (134 genes were upregulated and 86 genes were downregulated) by 180 genes (97 genes were upregulated and 83 genes were downregulated) from *Arabidopsis* genes (Supplementary Table 5 and Supplementary Figure 2). Among these genes, 60 genes in *Arabidopsis* and 72 genes in *Chlorella* were upregulated, such as *Ce. 6624* (*CAC3*), *Ce. 6305* (*BCCP2*), and *Ce. 1815* (*MOD1/ENR1*), which were key genes controlling fatty acid biosynthesis flux. Approximately 59% (95/160) of genes related to lipid metabolism annotated by MapMan were upregulated in the *AtLEC1* transgenic *Chlorella* strain compared with the WT. In transgenic *Arabidopsis* and *Chlorella*, overexpression of *LEC1* downregulated the expression of 39 genes in *Arabidopsis* and 45 genes in *Chlorella*, such as *Ce.59398* and *Ce.75003*, relative to protein biosynthesis, which were key genes controlling nitrogen metabolism (Supplementary Table 2).

Compared with overexpression of *LEC1* in *Arabidopsis*, some homologous genes had different changeable trends in *Chlorella*. The 41 genes in *LEC1* overexpression *Arabidopsis* were upregulated but not their 43 homologous genes in *AtLEC1* transgenic *Chlorella*, and their functions were involved in protein biosynthesis, vitamin metabolism and tetrapyrrole synthesis (Supplementary Table 2). The 26 genes were downregulated in *LEC1* overexpression *Arabidopsis*, but their homologous genes in *AtLEC1* transgenic *Chlorella* were upregulated (Supplementary Table 2). However, only 6 genes of *Chlorella* had the same regulation model with their homologous genes of *Arabidopsis* with the overexpression of *AtLEC1*. For example, *Ce.3222* and *Ce.7504* were significantly downregulated and could interact with Rubisco in the dark reaction of photosynthesis, whereas the other four genes *Ce.6081* (related to cell development), *Ce.7764* (ethanol dehydrogenase), *Ce.4276* (ATPase), and *Ce.6877* (aldehyde dehydrogenase) were significantly upregulated. The functions of the protein-modified related genes *Ce.1480* and *Ce.4858*, encoding Golgi body localization proteins, were contrary to their regulation in *Arabidopsis*. Thus, it would be of a great interest to dissect the differences in *AtLEC1* function in *Chlorella* and *Arabidopsis*.

We did not find *AtLEC1-Like* sequences in *C. ellipsoidea.* In the alignment of protein sequences between *AtLEC1* and other species, 14 homologs of *AtLEC1* were screened, and the similarities among them were 34.9–78.3%. The selected genes/proteins include *AtLEC1-like* from algae (MpuLEC1L, CsuLEC1L, MspLEC1L, OluLEC1L, CreLEC1L, VcaLEC1L, and DsaLEC1L), and higher plants (GmLEC1, BnLEC1, and AtL1L), and several NF-Y subunits (ScHAP323, HsNF-YB24, MmNF-YB38, and CeNF-YB) (Supplementary Table 6). All of these genes contain a conserved HFD domain (Nardone et al., 2017) (Supplementary Figure 3). *LEC1* in higher plants derived only by a suitable promoter can be used to improve the seed oil content (Mu et al., 2008; Tan et al., 2011). However, homologs of *LEC1* from the algae may improve the oilseed crop without abnormal agricultural traits through the genetic engineering due to no function differentiation for specific tissues as in higher plants, which warrants further investigation.

The *AtLEC1* could significantly increase the lipid content and decrease the protein content of *C. ellipsoidea* without affecting the growth rate of strains and biomass under mixotrophic culture and autotrophic culture conditions. Transcriptome sequencing analysis showed that *AtLEC1* could promote expressions of 59% of the genes related to the lipid biosynthesis in *C. ellipsoidea*, but the differences of mechanism of *AtLEC1* in regulating lipid accumulation in *C. ellipsoidea* and *Arabidopsis thaliana* warrants further investigation. In general, our research provides a new means of improving the lipid content in *Chlorella* and may help to elucidate the mechanism governing lipid accumulation in *Chlorella*.

## Data Availability Statement

The datasets presented in this study can be found in online repositories. The names of the repository/repositories and accession number(s) can be found in the article/Supplementary Material.

## Author Contributions

ZH and CF designed and supervised the study. XaL, DZ, and JZ performed the experiments. XaL, DZ, XuL, and CF analyzed the data. XaL and DZ wrote the manuscript. ZH, YC, YH, and CF revised the manuscript. RR-CW helped to direct the graduate research, interpreted and discussed data, and revised the manuscript. All authors have read and approved the manuscript.

## Conflict of Interest

The authors declare that the research was conducted in the absence of any commercial or financial relationships that could be construed as a potential conflict of interest.

## Footnotes

1 http://www.geneontology.org

2 https://bigd.big.ac.cn/databases

3 http://mapman.gabipd.org/home

## Supplementary Material

Supplementary Figure 1 *AtLEC1* regulated metabolism pathway.

Supplementary Figure 2 Venn diagram showing the differentially expressed genes in *AtLEC1* transgenic *C. ellipsoidea* and *A. thaliana*.

Supplementary Figure 3 Sequence analysis of LEC1 from different species.

Supplementary Table 1 Primers used for gene cloning, detection of genes by PCR and quantitative real-time PCR.

Supplementary Table 2 All the differentially expressed genes based on the transcriptomic analysis between the LEC1-1 strain and the wild-type strain.

Supplementary Table 3 Genes related to protein metabolism, RNA metabolism and transport in *AtLEC1* transgenic *C. ellipsoidea*.

Supplementary Table 4 *AtLEC1* regulated candidate genes related to protein and carbohydrate metabolism in the metabolic network.

Supplementary Table 5 Differentially expressed genes in *AtLEC1* transgenic *C. ellipsoidea* and *A. thaliana.*

Supplementary Table 6 Protein sequence homologous alignment of *AtLEC1* from different species.
