# Supplementary material for: Overexpression of the Transcription Factor AtLEC1 Significantly Improved the Lipid Content of Chlorella ellipsoidea
Source: Front Bioeng Biotechnol. 2021 Feb 17;9:626162. doi: 10.3389/fbioe.2021.626162 (PMC7925920; doi:10.3389/fbioe.2021.626162)
Supplement: Supplementary Figure 1 — AtLEC1 regulated metabolism pathway. [file Image_1.pdf]

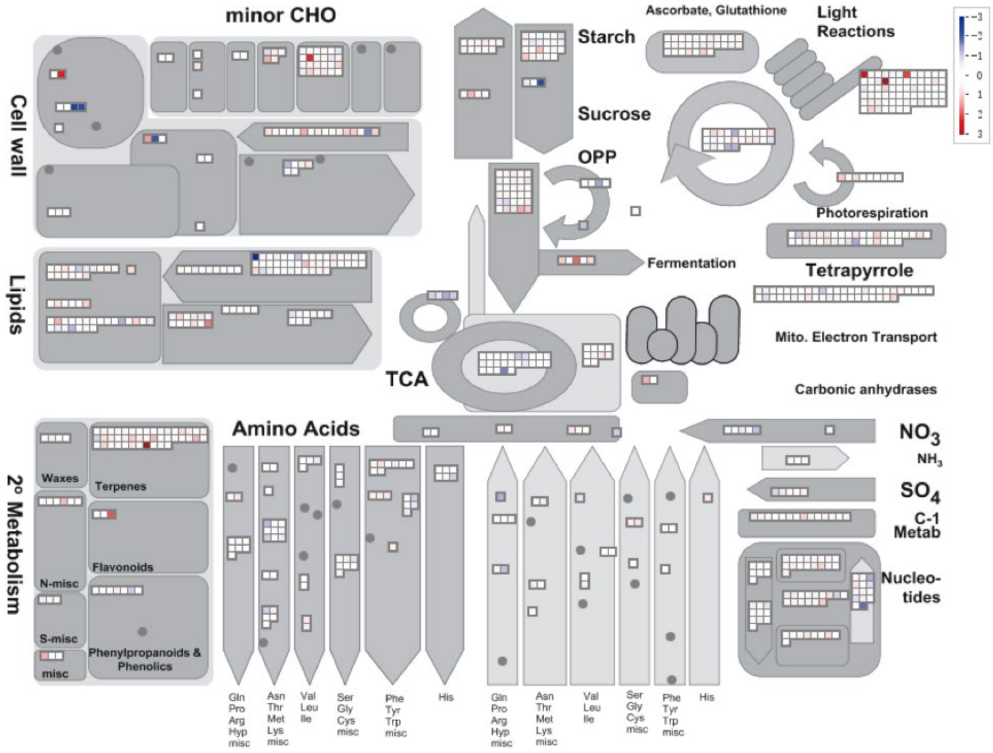

Supplemental Figure 1. ***AtLEC1*** **regulated metabolism pathway.**

Note: *AtLEC1* mediated metabolic network diagram showed that a total of 4,841 genes were annotated by *A. thaliana*. Figures in gray indicated different metabolic pathways, and small boxes indicated genes. Fill color of small boxes showed the fold Change in LEC1-1 vs WT. It was analyzed by MapMan.

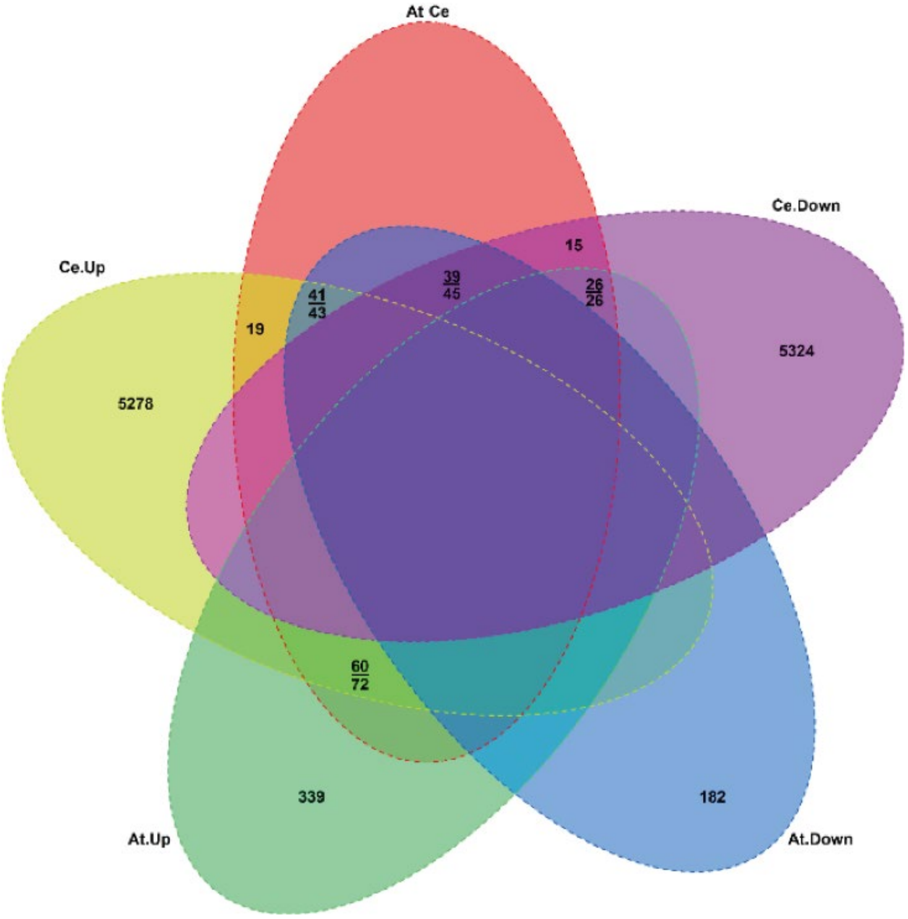

Supplemental Figure 2. Venn diagram showing the differential expression genes in *AtLEC1* transgenic *C. ellipsoidea* and *A. thaliana*.

Note: Ce, *Chlorella ellipsoidea*; At, *Arabidopsis thaliana*; Ce.up, upregulated in *AtLEC1* transgenic strains; Ce.down, downregulated in *AtLEC1* transgenic strains; At.up, upregulated in *A. thaliana*; At.down, downregulated in *A. thaliana*; the number above the bar represent the differential expression genes in *Arabidopsis* and the numbers below the bar represent the differential expression genes in *C. ellipsoidea*.

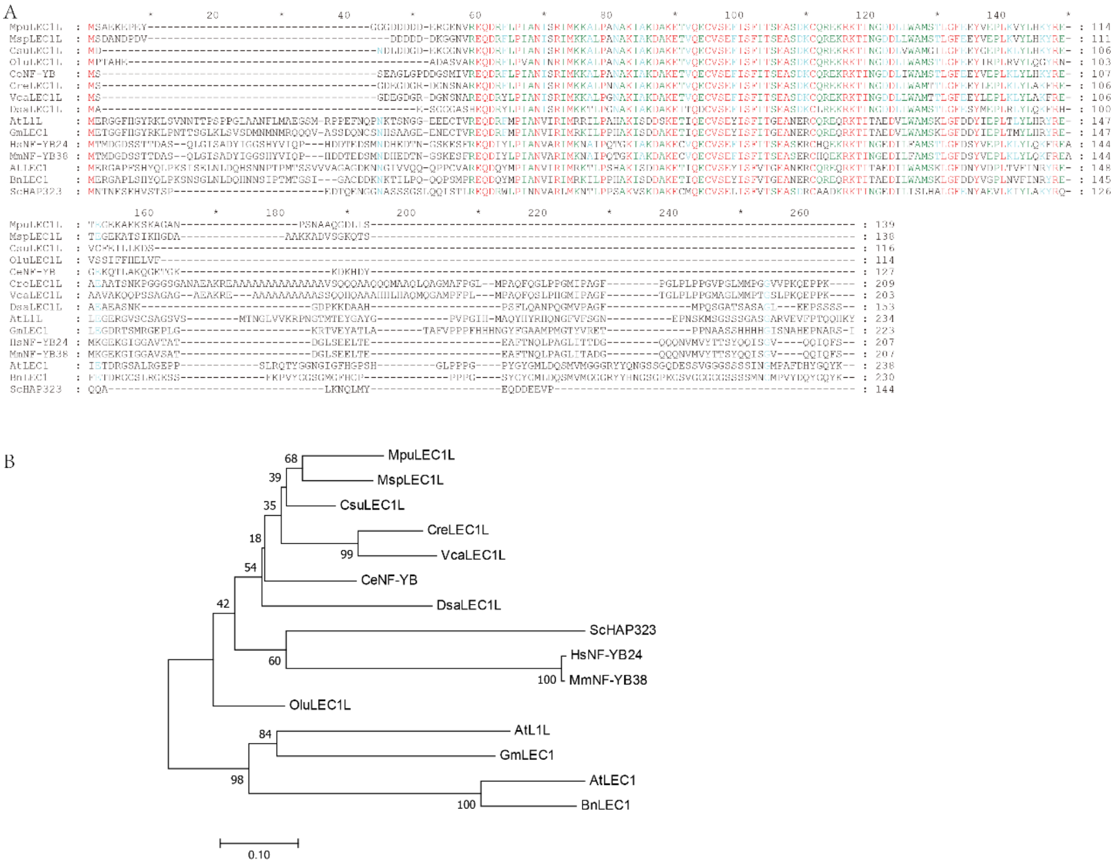

Supplemental Figure 3. Sequence analysis of LEC1 from different species.

Note: A, alignment of protein sequences. B, the phylogenic tree of LEC1. MpuLEC1L (*Micromonas pusilla*, accession no. C1N154), MspLEC1L (*Micromonas commode*, accession no.C1FEL2), CsuLEC1L (*Coccomyxa subellipsoidea*, accession no. I0Z9V0*)*, OluLEC1L (*Ostreococcus lucimarinus*, accession no. A4RSN4), CreLEC1L (*Chlamydomonas reinhardtii*, accession no.A0A0I9QPW6), VcaLEC1L (*Volvox carteri f. nagariensis*, accession no.D8TT52), DsaLEC1L (*Dunaliella salina*, accession no. ALA65390.1), AtLEC1 (*A. thaliana*, accession no. Q9SFD8), AtL1L (*A. thaliana*, accession no. Q84W66), ScHAP323 (*Saccharomyces cerevisiae*, accession no. CAA84840.1), HsNF-YB24 (*Homo sapiens*, P25208.2), MmNF-YB38 (*Mus musculus*, AAH10719.1), GmLEC1 (*Glycine max*, accession no. B5KMS8), BnLEC1 (*Brassica napus,* accession no. EU371726*)*. Letters in red, green and cyan, interval values of similarity were 100%, 70% and 50% at one site, respectively.

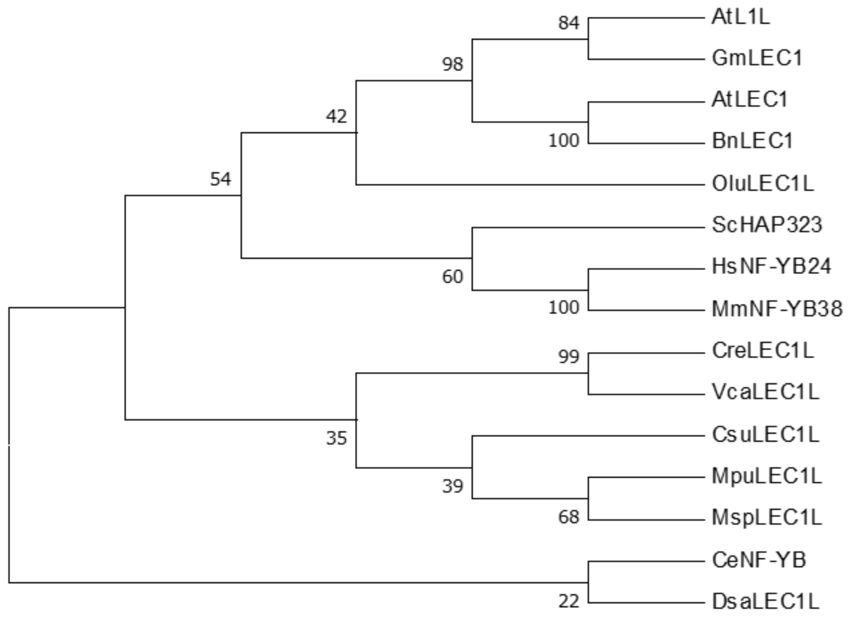

Supplemental Figure 4. Evolutionary relationships of *AtLEC1* from different species.

The evolutionary history was inferred using the Neighbor-Joining method [1]. The bootstrap consensus tree inferred from 1000 replicates [2] is taken to represent the evolutionary history of the taxa analyzed [2]. Branches corresponding to partitions reproduced in less than 50% bootstrap replicates are collapsed. The percentage of replicate trees in which the associated taxa clustered together in the bootstrap test (1000 replicates) are shown next to the branches [2]. The evolutionary distances were computed using the Poisson correction method [3] and are in the units of the number of amino acid substitutions per site. The analysis involved 15 amino acid sequences. All ambiguous positions were removed for each sequence pair. There were a total of 267 positions in the final dataset. Evolutionary analyses were conducted in MEGA7 [4].
